# Big Data for Drug Discovery: Some Historical Landscape, Considerations, and Applications for a Medicinal Chemist

**DOI:** 10.1021/acsomega.5c08828

**Published:** 2026-01-26

**Authors:** João A. L. de Lima, Lucas Silva Franco, Lídia M. Lima

**Affiliations:** Institute of Biomedical Science, 28125 Federal University of Rio de Janeiro, Avenue Carlos Chagas Filho 373, CCS, Block B Room B14 Rio de Janeiro, Rio de Janeiro 21941-902, Brazil

## Abstract

Big Data (BD) has the potential to transform the process of drug discovery. The integration of chemical, biological, pharmacological, and clinical information facilitates the expeditious conception of high-value projects, thereby enhancing the identification of hits and the generation of superior leads or repositioned candidates while concomitantly reducing time and costs. In this review, we demonstrate that BD extends beyond the scope of ligand discovery, thereby supporting the identification of novel pharmacological targets through the integration of genomic, proteomic, and metabolomic data sets. This integration adds further depth and guides the development of individualized therapies. When combined with combinatorial chemistry, high-throughput screening, and artificial intelligence (AI), BD expedites the identification of compounds that exhibit optimal pharmacokinetic and pharmacodynamic profiles. The impact of BD extends to later stages of drug development, including regulatory evaluation and clinical translation. This demonstrates that BD is no longer a supplementary tool but a cornerstone for rational molecular design, predictive modeling, and data-driven drug discovery. Although the benefits generated by the use of BD and AI in MedChem are evident, the impact of the widespread use of these data and tools raises a series of philosophical questions that need to be discussed since the popularization of large language models (LLMs) has resulted in the generation of promiscuous data, which, from a scientific point of view, lacks the criteria necessary for such data to be considered meaningful. All of these factors demonstrate the need for intentional dialogue on how these tools should be applied within the hermeneutics of biomedical sciences themselves, in order to ensure a lucid discussion on the nature of the method, harmonizing this apparent tension between human and AI, which has been a source of controversy since the exponential rise of ChatGPT and various other LLMs.

## Graphical abstract

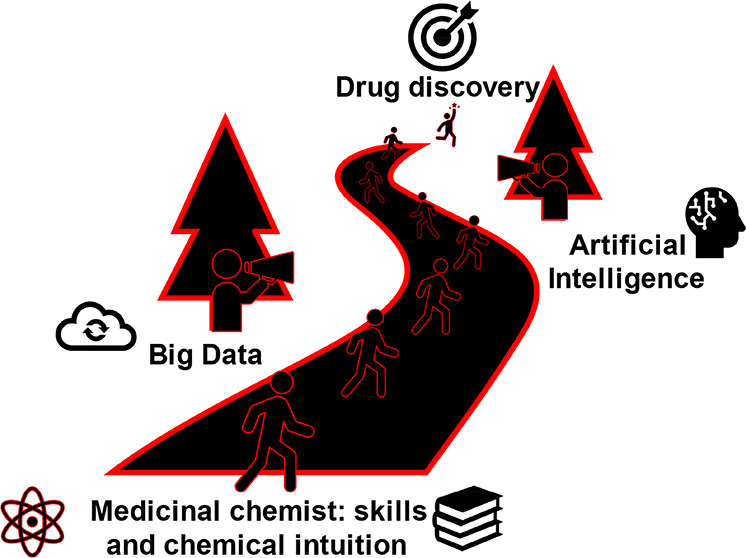

## Introduction

“Big Data” (BD) has become one of the most quoted terms in the field of data analysis in recent decades, and its use is now widespread in several areas of research.^1^ It is noteworthy that although the term BD was first introduced in 2005 by data scientist Roger Magoulas (Director of Market Research at O’Reilly Media), the use of a large amount of independent data to build models that respond to a need had already been reported in the middle of the 20th century (“Information Age”).^2^ According to records, the first public project related to BD was initiated in 1937, during Franklin Roosevelt’s administration in the United States. The project entailed the examination of social security contributions for a population exceeding 26 million Americans.

With the advent of the first data processing machine (Colossus) in 1943, a natural technological evolution, in terms of storing information, occurred. This development laid the foundational cornerstone for the subsequent development of contemporary computing machines.^3,4^ After the conclusion of the Second World War (1945) and the advent of the Cold War (1991), a significant proportion of the United States’ technological arsenal (comes from cryptographic analysis) had already attained the capacity to undertake large scale data collection and processing in an automated manner. This evidence suggests that a new paradigm concerning data storage was established at this point in time.^5–7^ Consequently, the utilization of BD has undergone substantial expansion, extending beyond the scope of social security analysis. Moreover, the genesis of information exchange through hypertext, otherwise known as the “World Wide Web”, a term conceptualized by computer scientist Tim Berners-Lee, promoted an exponential growth in the production and storage of networked data.^8^ Given these considerations, it can be assumed that BD exerts a direct influence on the manner in which data are stored, processed, and evaluated, with infrastructure capacity in all these stages always being taken into account.^9^

Therefore, BD can be defined as a large set of data (structured, semistructured, and unstructured) capable of being stored (in volume and variety of data) and processed at a speed that allows appropriate analysis and interpretation of the data, generating useful values for achieving the intended objectives of a data analysis project.^9^ Due to the conceptual complexity of BD, some hallmarks have been identified in an attempt to systematize their fundamental attributes. These hallmarks are systematized by the model of the four “V’s” (volume, variety, velocity, and veracity) or the five “V’s” (which adds the term value as an attribute as relevant as the others).^10^

In BD, “volume” is defined as the initial analysis of a machine’s capacity to store available data. It should be acknowledged that there is no pre-established quantitative limitation that is the standard for classifying these data as BD, since BD data ranges from peta (10^15^) to exabytes (10^18^), with this determination being relative to the available infrastructure and after preprocessing the data.^11^ “Variety”, on the other hand, refers to how these data are organized, namely, structured (CSV—comma separated value, XLS—excel spreadsheet, or SQL—structure query language), semistructured (XML—extensible markup language, JSON—JavaScript objective notation), and unstructured (audio, video, image, text, among others).^12^ Figure 1 illustrates the difference between data types.

**Figure 1:**
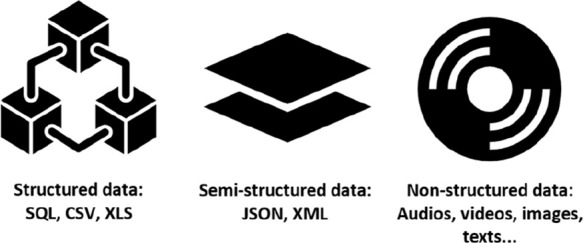
Types of data analyzed in a BD (Source: Author).

Another important attribute of BD is “veracity”, which refers to the precision, quality, and integrity of available data. These parameters are fundamental to ensuring the reliability of the data. Therefore, this attribute must be considered in BD protocols to distinguish which data to use and avoid false positives and those with low precision and accuracy.^13,14^

All these attributes are important for determining the “value”, which demonstrates the relevance of the data in answering a current demand. This is a fundamental step in establishing the significance and benefit of the data, and this attribute is pivotal for making decisions about whether to continue or discontinue a BD project.^15,16^ Figure 2 schematically shows the five main BD attributes.

**Figure 2:**
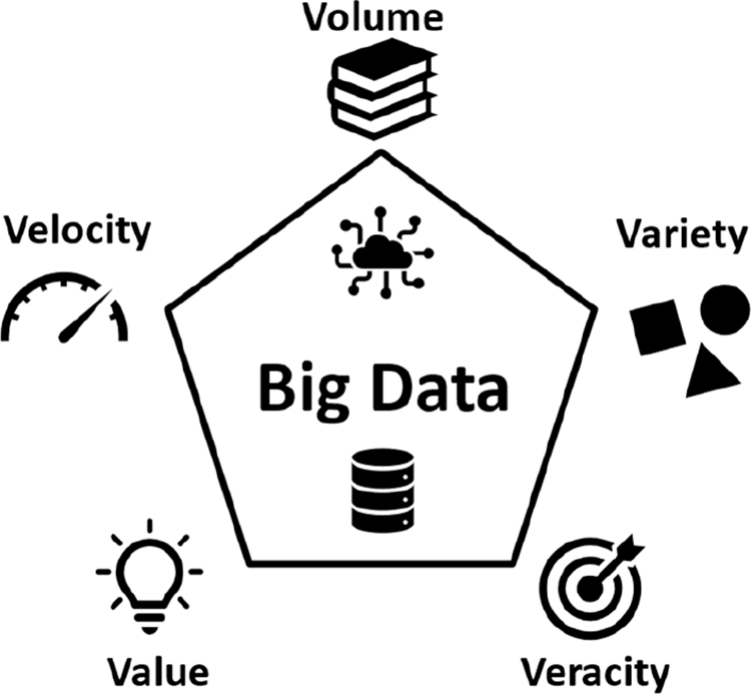
Five “Vs” (volume, variety, velocity, veracity, and value) are described as important attributes in BD (Source: Author).

## Historical Aspects of BD in Drug Discovery

Among the primary areas of BD application, the health sector stands out among the sciences that have been incorporating it the most in the various stages of diagnosis, prevention, and treatment of diseases.^17,18^ This high volume of information, derived from diverse types of chemical, genetic, biochemical, pathophysiological, and pharmacological tests (both preclinical and clinical trials), provides researchers the opportunity to explore and understand disease-related mechanisms, offering valuable material for subsequent BD project development aimed at prevention and treatment strategies.^19–22^ Public health organizations and institutions maintain platforms that disclose the availability of open-access data and facilitate the development of data-driven initiatives. In this sense, a myriad of institutions and databases provide results from genetic, biochemical, and biological studies. Table 1 illustrates some of the most frequently used web tools, organized by the purpose of each data set.

**Table 1:** Main Open-Source Platforms in the Health Area for BD Applications

European Bioinformatics Institute (EMBL-EBI)		
web-tool	function	reference
European Nucleotide Archive (ENA)	public archive for nucleotide sequence data	^193^
ArrayExpress	functional genomic data collection, stores data from high-throughput functional genomics experiments	^194^
Ensembl	genome browser for vertebrate genomes	^195^
UniProt	resource of protein sequence and functional information	^196^
PDBe	provides a high quality, up-to-date, and integrated resource of macromolecular structures	^197^
ChEMBL	curated database of bioactive molecules extracted from medicinal chemistry	^198^

National Center for Biotechnology Information (NCBI)		
web-tool	function	reference
GeneBank	genetic sequence database, an annotated collection of all publicly available DNA sequences	^199^
Taxonomy	curated database with classification and nomenclatures for all of the organisms with public sequences	^200^
Entrez	molecular biology database system for a rapidly searching sequence and bibliographic data	^201^
BLAST	online tool for Basic Local Alignment of nucleotide and proteins	^202^
Clinical Trials (CTG)	database of clinical research studies and information about their results	^203^
PubChem	largest database collection of freely accessible chemical information	^204^
PubMed	resource supporting the search and retrieval of biomedical and life sciences literature	^205^

The European Bioinformatics Institute (EMBL-EBI), officially established in 1992, was the world’s first large database of nucleotide sequences. Created by researchers at the European Molecular Biology Laboratory in Heidelberg, Germany, the approach aimed to facilitate the search for and deposit of DNA sequences on a single platform. Due to the extensive amount of data provided by geneticists from all over Europe, the database soon became a source for comparative data analysis, sparking interest from the commercial sector in using this platform to build large-scale genomic projects.

The establishment of the Sanger Sequencing Institute led to the relocation of EMBL-EBI to the United Kingdom, where it remained in Hinxton, connecting the database to the protein sequence data supplied by the Swiss-Prot-TrEMBL platform (henceforth Uniprot). Since then, EMBL-EBI has been essential for any project correlating biological data with the life sciences.^23–25^

Unlike EMBL-EBI, the National Center for Biotechnology Information (NCBI), created in 1988, was developed by three independent American organizations: genomic researchers on Capitol Hill; members of the National Library of Medicine (NLM/NIH); and members of the Committee on Aging, who presented Bill HR393 in the House of Representatives in 1987. Despite their autonomy in different spheres of sovereignty, these organizations shared the same objectives. This resulted in the merger of Bill HR393 with the NIH Reauthorization Bill (Bill 100–607).^26^

Presently, the NCBI-NIH stands as the most sizable biomedical data repository on the planet, comprising an array of over 35 databases, which collectively encompass more than 3 billion data elements. Much of the information provided by NCBI is complementary to the information made available by EMBL-EBI. For instance, GenBank, the genomic data platform equivalent to the European nucleotide archive, provides the genetic information necessary to construct the sequences offered by UniProt. BLAST enables the alignment-based comparison of the various types of homologous sequences offered by UniProt, making it a valuable tool for correlating genomic and proteomic data.^27^

In this context, the pharmaceutical sector is distinguished by its consistent utilization of this information [clinical data, electronic health records (EHRs), omics data (genomics, transcriptomics, proteomics, metabolomics), epidemiological data, chemical data, image data, pharmacovigilance reports, and scientific literature] as input data. Indeed, when data are integrated, it logically constitutes a BD problem. This is much more feasible with BD projects built directly by the drug industry, which have a considerable technological arsenal and can develop these projects through companies that specialize in building BD projects in drug discovery.^28,29^

## Fundamentals of BD in Drug Discovery and Development

In drug discovery, the use of BD involves collecting chemical, biological, pharmacological, and clinical data. These data serve as parameters for designing fast, robust, high-value projects that are characterized by complexity and heterogeneity. BD projects in the early stages of pharmaceutical research support decision-making by identifying promising bioactive molecules, hits, prototypes, and drug candidates.^29^

These approaches enhance the identification rate of validated hits, contributing to the development of higher-quality leads or repositioned candidates for specific diseases. Ultimately, this reduces the time and costs associated with drug discovery and development. Furthermore, BD projects carried out in discovery and development are not exclusively restricted to the discovery of ligands. This broadens the scope of research in the search for potential pharmacological targets, a promising BD strategy.^30^ For this reason, the following topic discusses some important biological sources.

## Relevant Data Sources: Genomics, Proteomics, Metabolomics

Studies involving the human genome are among the earliest sources of BD projects. This is due to the exponential increase in data following the completion of the Human Genome Project, as well as advancements in sequencing technologies, such as next-generation sequencing, whole-genome sequencing, and whole-exome sequencing.^31,32^ Genomic information has established a new paradigm for analyzing data based on the genetic code. This allows scientists to take the first steps toward developing personalized therapies. A significant component of this strategy involves the use of EHRs, which, when combined with genetic information, enable the correlation of diseases and genetic disorders. A 2017 survey by He and Co-workers shows more than 12 projects combining EHRs with genomic data, most of which provide free access to this information.^31–35^

Although genetic information is fundamental to any BD study, biomarkers related to the characteristics of each disease are also necessary for the drug discovery process. The comprehensive evaluation of this phenomenon necessitates the utilization of phenotypic data, with proteins serving as a critical “substrate” in this scientific inquiry. It is notable that proteomic methodologies have attained heightened prominence within the domain of medical sciences on a global scale.^34^

Due to the necessity of correlating genotypic and phenotypic data, there is an imperative for proteomic data to be made available in open-access database repositories. This will provide a scientific framework to support the conclusions made in the academic publications. The resulting phenomenon of significant proteomic data abundance in public archives has materialized. A notable repository facilitating this exchange is ProteomeXchange, an eminent consortium of proteomic resources that has spearheaded the unification of data submission and distribution processes involving mass spectrometry.^34,35^

However, the expansion of proteomics as a data source carries direct implications for the ethical and legal domains since these data pertain to information concerning the clinical state of human patients. The pertinence of this issue has led to the establishment of legal constraints on the use of these data in both North American and European jurisdictions. These restrictions mandate that researchers apply for what is termed “controlled access” data.

This legal issue ultimately proves to be a challenge in the utilization of proteomics for the development of BD projects in the domain of drug discovery, necessitating the employment of alternative sample types.^36^ In this regard, metabolomics emerges as a good option due to its emphasis on the characterization of small endogenous molecules (avoiding ethical and legal constraints), with this kind of data being very useful in the medical sciences, particularly within the domain of MedChem. Since it addresses a broad array of biomolecules that vary in their physicochemical properties, a characteristic that differentiates it from proteomics, which concentrates on macromolecules, a multitude of analysis strategies (including spectroscopic techniques, such as nuclear magnetic resonance, and spectrometric methods, such as mass spectrometry) are employed. These strategies yield both qualitative and quantitative data, which is highly advantageous in the context of BD.^37^

The advent of novel techniques, such as metabolite imaging, coupled with the advancement of underlying technologies, has led to a marked surge in the utilization of metabolomics in biomedical and pharmaceutical applications. In the context of BD for drug discovery, metabolomics has been instrumental in the development of individualized therapy strategies. These strategies are based on data related to personalized phenotypic profiles and real-time monitoring of the impacts that administered drugs have on the metabolic profile.^38,39^

The application of metabolomics in the development of targeted antitumor therapies has been demonstrated, with these therapies being based on the phenotypic profiles of cancer patients. Numerous online platforms host metabolomic data, which, when integrated, yield valuable insights for developing treatment strategies. In this regard, BD projects play a pivotal role in this process by facilitating the translation of complex metabolic information into actionable pharmacological interventions.^39,40^ One of the applications of BD based on these data provided is in the strategy called pharmacometabolomics, which integrates data on the various metabolites generated after application (in vitro) or administration (in vivo) of bioactive substances.^41^ These metabolites are subsequently classified in order to comprehend their therapeutic (prognostic, diagnostic, predictive, and treatment markers) or deleterious (monitoring, safety, and susceptibility markers) impacts. Furthermore, the application of pharmacometabolomics offers a distinct advantage in the construction of “metabotypes,” which is defined as a comprehensive synthesis of the stratified individual results in relation to the responses generated after administration of the drug.^42,43^ Figure 3 shows a representative pharmacometabolomic workflow.

**Figure 3:**
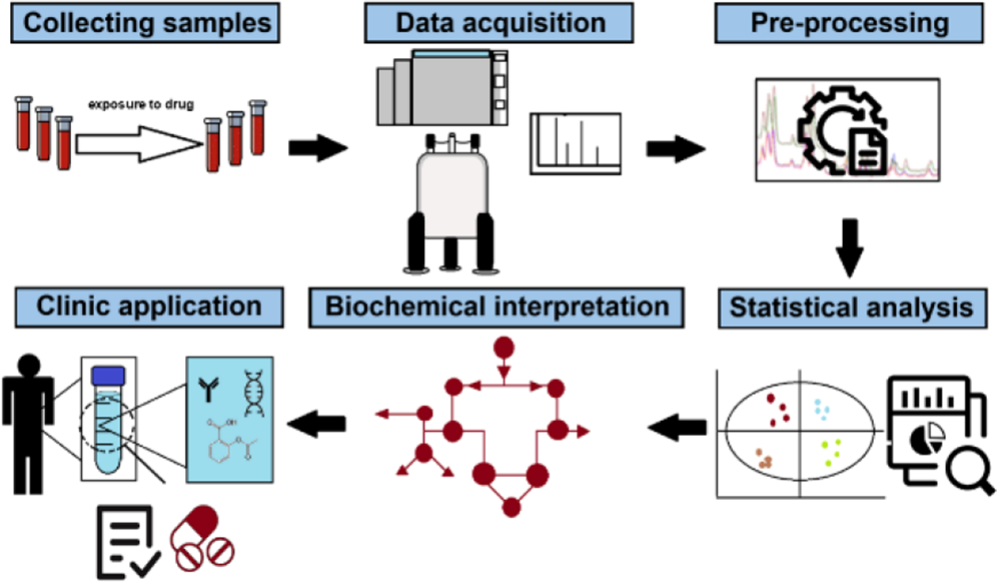
Pharmacometabolomic workflow (Adapted from Amaro et al. and reproduced according to terms and conditions of CC BY 4.0 (https://creativecommons.org/licenses/by/4.0/).^44^

## Relevant Data Sources: Chemical and Biological

The application of BD for drug discovery has also shown considerable promise in the analysis of data derived from the integration of results generated through combinatorial chemistry with robotics and high-throughput screening (HTS). These data have profoundly impacted the manner in which academia and the pharmaceutical industry conduct their drug discovery initiatives. It facilitates expedited screening of a vast number of compounds, ranging from thousands to millions, that possess desirable pharmacokinetic and pharmacodynamic properties.^44^ Advancements in gene editing, three-dimensional cell culture integrated with HTS, and microsystems with electronic devices inserted directly into organs have also amplified the understanding of the effects of pharmacotherapeutic substances on the cellular micro and macro-environment, providing valuable information for drug planning from BD projects.^44^

Due to the inherent complexities of chemical and biological data in the context of BD for drug discovery, other attributes related to the five Vs model have been added: validity, vocabulary, venue, visualization, and volatility. It is important to note that these attributes are, in fact, subattributes derived from the five Vs previously mentioned: volume, variety, velocity, veracity, and value.^44^ Therefore, given the additional attributes’ capacity to introduce further complexity to database data, it becomes imperative within any given protocol to undertake a thorough evaluation of the standardization of terminology (i.e., vocabulary) across all sample data sets and disparate files.

This evaluation must encompass both intraset standardization and interfile standardization, underscoring the critical need for comprehensive standardization of these data during the preprocessing stage. Furthermore, if subsequent verification across varied platforms (i.e., venue) substantiates this necessity, prompt action must be taken to implement these standardization measures. Given the substantial number of testing protocols, the standardized data must be evaluated for its representativeness, and the “Validity” stage is pivotal for determining the veracity of the data. The construction of the results necessitates the graphical visualization of all contributing factors to facilitate data interpretation. In this manner, the “visualization” stage permeates all the phases and is of fundamental importance in determining whether the data introduced into the analysis are significant or whether they have already lost their relevance (considering the objectives to be achieved) and can therefore be considered “volatile” (volatility) and consequently with an inappropriate value (i.e., Value).^45^

Figure 4 shows all of these new attributes. It is important to mention that underestimating these attributes can compromise the success of the process. Fortunately, many of these challenges are mitigated by modern database platforms, which provide an integrated and comprehensive set of physical-chemical and biological data on bioactive compounds, enabling decision-making with higher success rates. Therefore, before starting data analysis, a critical step in a project is the selection of the appropriate platform(s). This choice is typically guided by predefined criteria aligned with the specific stage of the drug research pipeline, whether in the discovery phase (involving initial exploration of ligands, hits, prototypes) or in the development phase (covering preclinical and clinical studies).^46^

**Figure 4:**
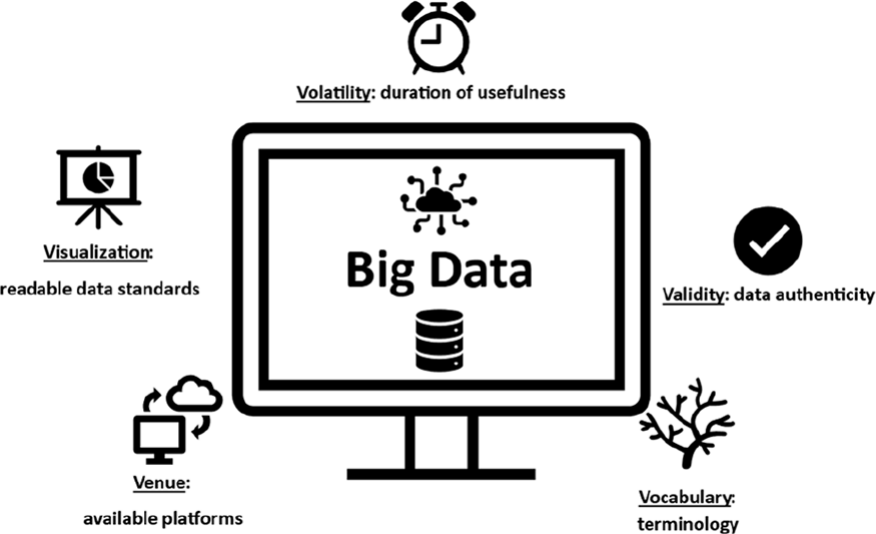
Expansion of the five Vs model of BD in the context of drug discovery (Source: Author).

## How Integrating Data from Different BD Sources Can Generate Valuable Insights for Medicinal Chemists

To understand whether there is, in fact, a relation between the use of BD and the construction of MedChem projects, a survey was conducted of papers indexed in the academic databases Web of Science (Clarivate Analytics) and SCOPUS (Elsevier). The query was performed within the field of MedChem, encompassing research, review, communication, and opinion documents, among others. The search considered documents published between the years 2013 and 2025 that contained the keywords “BD” and “Medicinal Chemistry”. A total of 108 documents were found. After removing duplicates (29 excluded from Scopus) and considering the inadequate content present in each work (21 excluded from Scopus and 8 from Web of Science, with these analyses being performed by direct inspection), 50 documents were selected. In addition, to obtain more documents that were not indexed in these two databases, we used the same keywords in Google’s search tool (considering if these keywords would be the main theme of these documents), where 39 documents were found. It is illustrated in Chart 1 and outlined in Table 2. A prism diagram, which is available at the conclusion of this article, provides a comprehensive description of the search methodology.

**Chart 1:**
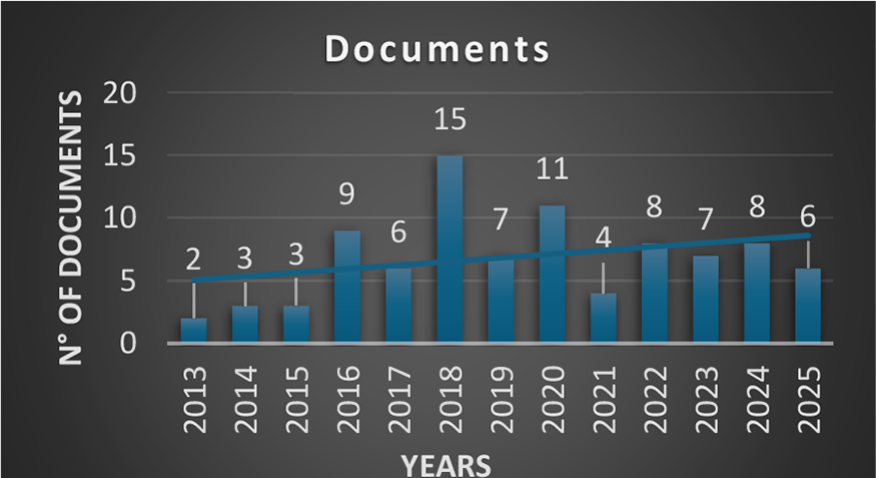
Number of Publications Found on Scopus, Web of Science, and Google Scholar Platforms Using Both Keywords: “Big Data” and “Medicinal Chemistry”. Source: Chart Created Using Excel Software v. 25.11.

**Table 2:** List of Works Found in the Literature That Describe and Recommend the Applications of BD in the Context of MedChem^a^

title of the article	approach/design	references
Matched molecular pair analysis in drug discovery	LBDD	Dossetter; Griffen; Leach, 2013 ^53^
Research on Pharmaceuticals Patents in Times of Big Data: A Contribution of the Web 2.0 for Medicinal Chemistry	DDDD	Magalhães et al., 2013 ^71^
“Big data” in pharmaceutical science: Challenges and opportunities	FBDD, LBDD, SBDD, PDD	Dossetter et al., 2014 ^126^
Data-driven medicinal chemistry in the era of big data	FBDD, LBDD, SBDD, PDD, DDDD	Lusher et al., 2014 ^127^
Computational target fishing: what should chemogenomics researchers expect for the future of in silico drug design and discovery?	LBDD, SBDD	Wang; Xie, 2014 ^55^
Progress in visual representations of chemical space	LBDD	Osolodkin et al., 2015 ^128^
Medicinal chemistry in the era of big data	FBDD, LBDD, SBDD, PDD, DDDD	Richter; Ecker, 2015 ^129^
Finding the right approach to big data-driven medicinal chemistry	DDDD	Lusher; Ritschel, 2015 ^130^
Big Data Opportunities in Medicinal Chemistry: how can patents in dengue affairs contribute?	DDDD	Magalhães et al., 2016 ^72^
Big Data from Pharmaceutical Patents: A Computational Analysis of Medicinal Chemists Bread and Butter	DDDD	Schneider et al., 2016 ^73^
Cancer genomics: opportunities for medicinal chemistry?	LBDD, SBDD, PDD	Wang; Xie, 2016 ^131^
Does “Big Data” exist in medicinal chemistry, and if so, how can it be harnessed?	FBDD, LBDD, SBDD, PDD	Tetko; Engkvist; Chen, 2016 ^49^
BIGCHEM: Challenges and Opportunities for Big Data Analysis in Chemistry	FBDD, PDD, DDDD	Tetko et al., 2016 ^132^
Impact of binding site comparisons on medicinal chemistry and rational molecular design	LBDD, SBDD	Ehrt; Brinkjost; Koch, 2016 ^133^
Brain-inspired cheminformatics of drug-target brain interactome, synthesis, and assay of TVP1022 derivatives.	LBDD	Romero-Durán et al., 2016 ^134^
A perspective on implementing a quantitative systems pharmacology platform for drug discovery and the advancement of personalized medicine	PDD	Stern et al., 2016 ^54^
Minimizing Bias in Target Selection by Exploiting Multidisciplinary Big Data and the Protein Interactome	PDD	Al-Lazikani; Workman, 2016 ^135^
What Can We Learn from Bioactivity Data? Chemoinformatics Tools and Applications in Chemical Biology Research	FBDD, LBDD, SBDD, PDD, DDDD	Humbeck; Koch, 2017 ^136^
Best Practices of Computer-Aided Drug Discovery: Lessons Learned from the Development of a Preclinical Candidate for Prostate Cancer with a New Mechanism of Action	CADD	Ban et al., 2017 ^137^
Entering the “big data” era in medicinal chemistry: Molecular promiscuity analysis revisited	DDDD, PDD	Hu; Balorath, 2017 ^138^
Chemical Topic Modeling: Exploring Molecular Data Sets Using a Common Text-Mining Approach	DDDD	Schneider et al., 2017 ^139^
Macromolecular target prediction by self-organizing feature maps	SBDD	Schneider; Schneider, 2017 ^140^
Pharmacological property optimization for allosteric ligands: A medicinal chemistry perspective	LBDD	Johnstone; Albert, 2017 ^56^
Medicinal chemistry in drug discovery in big pharma: past, present and future	FBDD, LBDD, SBDD, PDD, DDDD	Campbell; Macdonald; Procopiou, 2018 ^120^
Can we accelerate medicinal chemistry by augmenting the chemist with Big Data and artificial intelligence?	FBDD, LBDD, SBDD, PDD, DDDD	Griffen et al., 2018 ^141^
Foundations of data-driven medicinal chemistry	DDDD	Bajorath, 2018 ^142^
Ask the experts: Computational chemistry	FBDD, LBDD	Matta; Hutter, 2018 ^143^
CHIPMUNK: A Virtual Synthesizable Small-Molecule Library for Medicinal Chemistry, Exploitable for Protein–Protein Interaction Modulators	LBDD, PDD, SBDD	Humbeck et al., 2018 ^144^
Perturbation Theory/Machine Learning Model of ChEMBL Data for Dopamine Targets: Docking, Synthesis, and Assay of New l-prolyl-l-leucyl-glycinamide Peptidomimetics	LBDD, SBDD, PDD	Costa et al., 2018 ^145^
Computational prediction of chemical reactions: current status and outlook	CASD	Engkvist et al., 2018 ^146^
Identification of Bioactive Scaffolds Based on QSAR Models	LBDD	Nakagawa; Miyao; Funatsu, 2018 ^75^
Data analytics and deep learning in medicinal chemistry	LBDD, SBDD, PDD	Bajorath, 2018 ^147^
PTML combinatorial model of ChEMBL compounds assays for multiple types of cancer	LBDD, SBDD, PDD	Bediaga; Arrasate; González-Díaz, 2018 ^148^
A review of ligand-based virtual screening web tools and screening algorithms in large molecular databases in the age of big data	CADD	Banegas-Lunas; Cerón-Carrasco; Pérez-Sanchez, 2018 ^74^
Evaluation of Kinase Inhibitor Selectivity Using Cell-based Profiling Data	DDMC, PDD	Miljković; Bajorath, 2018 ^57^
Recent applications of machine learning in medicinal chemistry	LBDD, SBDD, PDD	Panteleev; Gao; Jia, 2018 ^149^
Recent Advances in the Development of Pharmaceutical Agents for Metabolic Disorders: A Computational Perspective	PDD	Janardhan et al., 2018 ^150^
Parallelization of Molecular Docking: A Review	SBDD	Dong et al., 2018 ^151^
Multioutput Perturbation-Theory Machine Learning (PTML) Model of ChEMBL Data for Antiretroviral Compounds	LBDD, SBDD, PDD	Vásquez-Domínguez et al., 2019 ^152^
Advances and Perspectives in Applying Deep Learning for Drug Design and Discovery	LBDD, SBDD	Lipinski et al., 2019 ^82^
A Structure-Based Drug Discovery Paradigm	CADD	Batool; Ahmad; Choi, 2019 ^153^
ADMET modeling approaches in drug discovery	LBDD	Ferreira; Andricopulo, 2019 ^154^
Computational Drug Repurposing: Current Trends	PDD, DDDD	Karaman; Sippl, 2019 ^155^
Identifying Cancer Targets Based on Machine Learning Methods via Chou’s 5-steps Rule and General Pseudo Components	SBDD	Liang et al., 2019 ^156^
Pros and cons of virtual screening based on public “Big Data”: In silico mining for new bromodomain inhibitors	LBDD	Casciuc et al., 2019 ^157^
Drug Research Meets Network Science: Where Are We?	LBDD, SBDD, PDD	Recanatini; Cabrelle, 2020 ^158^
Toward reproducible computational drug discovery	DDDD	Schaduangrat et al., 2020 ^52^
PTML Model of ChEMBL Compounds Assays for Vitamin Derivatives	LBDD, PDD	Santana et al., 2020 ^159^
Big data science at AMED-BINDS	SBDD	Nakamura, 2020 ^160^
Advancing computer-aided drug discovery (CADD) by big data and data-driven machine learning modeling	CADD	Zhao et al., 2020 ^45^
QSAR without borders	LBDD	Muratov et al., 2020 ^83^
Ligand- and Structure-Based Drug Design and Optimization using KNIME	FBDD, LBDD, SBDD, PDD, DDDD	Mazanetz; Goode; Chudyk, 2020 ^161^
Heart Rate Variability Based Prediction of Personalized Drug Therapeutic Response: The Present Status and the Perspectives	PDD	Pei et al., 2020 ^162^
Big Data in Predictive Toxicology: Challenges, Opportunities and Perspectives	PDD	Richarz, 2020 ^163^
Data sets and their influence on the development of computer assisted synthesis planning tools in the pharmaceutical domain	CASD	Thakkar et al., 2020 ^164^
“Ring Breaker”: Neural Network Driven Synthesis Prediction of the Ring System Chemical Space	CASD	Thakkar et al., 2020 ^165^
Combinatorics, Big Data, Neural Network & AI for Medicinal Chemistry & Drug Administration	LBDD, SBDD, PDD, CADD, DDDD	Balasubramanian, 2021 ^166^
Evolving scenario of big data and Artificial Intelligence (AI) in drug discovery	LBDD, SBDD, PDD, CADD, DDDD	Tripathi et al., 2021 ^48^
State-of-the-art of artificial intelligence in medicinal chemistry	LBDD, SBDD, PDD	Bajorath, 2021 ^167^
A decade of machine learning-based predictive models for human pharmacokinetics: Advances and challenges	LBDD, PDD	Danishuddin et al., 2021 ^168^
Accelerating big data analysis through LASSO-random forest algorithm in QSAR studies	LBDD	Motamedi et al., 2022 ^80^
Machine Learning in Chemoinformatics and Medicinal Chemistry	CADD	Rodríguez-Pérez; Miljković; Bajorath, 2022 ^169^
Yes SIR! On the structure–inactivity relationships in drug discovery	LBDD	López-López; Gortari; Medina-Franco, 2022 ^170^
A Consensus Compound/Bioactivity Data set for Data-Driven Drug Design and Chemogenomics	DDDD	Isigkeit; Chaikuad; Merk, 2022 ^171^
From traditional to data-driven medicinal chemistry: A case study	LBDD, SBDD, PDD, CADD, DDDD	Kunimoto; Bajorath; Aoki, 2022 ^86^
Machine Learning in Antibacterial Drug Design	LBDD, SBDD	Jukič; Bren, 2022 ^84^
Chemspace Atlas: Multiscale Chemography of Ultralarge Libraries for Drug Discovery	CADD	Zabolotna et al., 2022 ^172^
Machine Learning and Artificial Intelligence: A Paradigm Shift in Big Data-Driven Drug Design and Discovery	DDDD	Pasrija et al., 2022 ^33^
Artificial intelligence and machine-learning approaches in structure and ligand-based discovery of drugs affecting central nervous system	LBDD, SBDD, PDD, CADD, DDDD	Gautam et al., 2023 ^173^
Specific contributions of artificial intelligence to interdisciplinary life science research – exploring and communicating new opportunities	DDDD	Bajorath, 2023 ^174^
Docking-based generative approaches in the search for new drug candidates	CADD	Danel et al., 2023 ^175^
New avenues in artificial-intelligence-assisted drug discovery	LBDD, SBDD	Cerchia; Lavecchia,, 2023 ^176^
Artificial Intelligence and Machine Learning Technology Driven Modern Drug Discovery and Development	LBDD, SBDD	Sakar et al., 2023 ^177^
Machine learning in Alzheimer’s disease drug discovery and target identification	LBDD, SBDD	Geng, Wang, Tang, 2023 ^85^
Yin-yang in drug discovery: rethinking de novo design and development of predictive models	CADD, LBDD, SBDD	Chávez-Hernández, López-López, Medina-Franco, 2023 ^178^
Generative AI: A systematic review using topic modeling techniques	LBDD, SBDD	Gupta et al., 2024 ^179^
Toward structure–multiple activity relationships (SMARts) using computational approaches: A polypharmacological perspective	PDD	Lopez-López; Medina-Franco, 2024 ^180^
Drug Repositioning and Artificial Intelligence: Is It a Promising Approach to be Used for Neglected Diseases?	LBDD, SBDD	Bongiovani et al., 2024 ^181^
Research on targeted drug design based on computer technology	CADD, LBDD, SBDD	Xu, 2024 ^182^
Application progress of deep generative models in de novo drug design	LBDD	Liu et al., 2024 ^183^
Big Data and AI in Natural Product Drug Discovery: Uncovering Hidden Medicinal Chemistry Gems	LBDD, DDDD	Panigrahi et al., 2024 ^184^
Experimental and computational models to understand protein–ligand, metal–ligand and metal-DNA interactions pertinent to targeted cancer and other therapies	SBDD	Patil et al., 2024 ^185^
Drug Discovery and Development: The Role of Artificial Intelligence in Drug Repurposing	DDDD	Aljofan; Gaipov, 2024 ^186^
Big data in computational medicinal chemistry, pharmacology and toxicology: Challenges and opportunities	LBDD, SBDD, PDD, CADD, DDDD	Shah; Ishfaq, 2025 ^187^
Big Data and Artificial Intelligence in Drug Discovery for Gastric Cancer: Current Applications and Future Perspectives	PDD	Nguyen; Tran; Le, 2025 ^188^
The Future of Medicine: AI and ML Driven Drug Discovery Advancements	CADD, DDDD	Patel et al., 2025 ^189^
Psychedelic Drugs in Mental Disorders: Current Clinical Scope and Deep Learning-Based Advanced Perspectives	PDD	Kim et al., 2025 ^190^
Chemical space visual navigation in the era of deep learning and Big Data	LBDD	Sosnin, 2025 ^191^
How generative Artificial Intelligence can transform drug discovery	LBDD, SBDD, PDD, CADD, DDDD	Jusoh et al., 2025 ^192^

a CADD—Computer-Assisted Drug Design; CASD—Computer-Assisted Synthesis Design; DDDD—Data-Driven Drug Design; FBDD—Fragment-Based Drug Design; LBDD—Ligand-Based Drug Design; PDD—Phenotypic Drug Design; SBDD—Structure-Based Drug Design.

This finding indicates that the term BD, like other fields, has been increasing in popularity in works directly related to MedChem over time. This increasing popularity can be attributed to the recognition of BD’s potential to enhance various stages of the drug discovery process. For medicinal chemists, access to extensive, integrated data sets supports the design, synthesis, and biological evaluation of bioactive molecules. Furthermore, since the complexity of therapeutic targets and chemical space increases, the use of BD tools has become imperative for the navigation and extraction of meaningful insights from high-dimensional data. In the following section, a systematic overview of the main approaches identified (Table 2) regarding the application of BD in MedChem will be presented. This discussion encompasses both classical drug design principles and emerging conceptual frameworks that have been identified in the extant literature. However, before the findings are analyzed and interpreted, it is essential to define some key criteria that characterize an effective integration of BD into MedChem workflows.

## Practical Applications of the Use of BD for Drug Discovery in the Context of MedChem

To be considered a BD project, a drug discovery pipeline must generally be evaluated through a multifaceted lens, encompassing a variety of joint approaches. Notably, this is the point raised by data scientists who argue whether the categorization of the application of virtual small molecule data in classical MedChem approaches can be considered as a BD project. However, when considered as a whole, these data elements can be regarded as a BD project since these approaches include the use of molecular descriptors (e.g., fingerprints), cross-references between chemical databases (e.g., PubChem and ChEMBL), biochemical, cellular, genomic, and proteomic assays, and clinical data (e.g., EHR). Another salient criterion pertains to the virtual chemical space that is to be investigated prior to determining the molecules to be selected for synthesis and subsequent analysis of their physicochemical and biological properties. Considering the aforementioned factors, the decision-making process requires high-dimensional analysis; together, these elements can also be considered a BD project.^47–49^

In this sense, there are many companies that use databases in their projects, with open repositories, that are widely used by scientific community and should be useful for using for MedChem projects based on BD, in which one of paper found describe (which is highly recommended for further reading) a list of the largest public cloud infrastructure service providers, software and packages that implements an automated, selected GitHub URLs of researchers working in the domain of computational drug discovery and web applications for handling various bioinformatic and cheminformatic tasks belonging to either a ligand-based or structure-based drug design (SBDD) approach.^52^

Since the article cited above already provides a compendium of pertinent examples and a thorough examination of the advantages and disadvantages of BD applied to MedChem, the present review aims to furnish a more comprehensive and descriptive discussion of the extant data. It will do so by accentuating certain points that have received insufficient attention in the existing literature. For this purpose, the discussion about the survey of papers highlighted in Table 2 is a comprehensive discourse that will ensue, elucidating the points reported in these works, glimpsing to underscore the aspects that promote an organic dialogue between BD and MedChem.

## Artificial Intelligence in Drug Discovery

First, most documents in the present survey illustrate a substantial proportion of the applications of BD in the domain of MedChem, employing artificial intelligence (AI) as their principal instrument during all phases of the drug discovery process (see Table 2). This result is consistent with the findings of recent studies by Catacutan et al. (2024)^50^ and Xu (2024)^51^, which provide compelling examples of artificial intelligence in drug discovery (AIDD) contributions in both preclinical and clinical trials. It is important to mention that, although some of the works identified in our research did not explicitly outline the use of AI methodologies in their platforms, software, or servers, they nevertheless discussed that such projects were employed by AI algorithms.^51–55^

So, this observation suggests the existence of a certain “symbiosis” between BD and AI when utilized in the context of AIDD.^56^ This phenomenon is due to a characteristic common to all types of AI, namely, the need for input data for machine learning, which is a fundamental attribute for defining the concept of AI. A direct implication that gives us a good understanding of how AI works and its relationship with data is the fact that all AI will always need data, but data will not always need AI.^57^ However, when this relationship is in the field of BDs, their dependence on machine learning algorithms to evaluate these data in a timely manner is so high that experts claim that the relationship between AI and BD implies a data paradox, so that AI needs data just as BD needs AI.^58–62^ Therefore, based on these considerations, we will now briefly discuss the main AI algorithms applied to BD projects built along the lines of MedChem.

## Supervised and Unsupervised Machine Learning Approaches in Drug Discovery

Machine learning, a subset of AI, involves the development of computational models capable of identifying patterns and making predictions from data without relying on explicitly programmed rules. To function effectively, ML requires both high-quality data sets and adequate computational infrastructure for data processing and storage. ML approaches are typically categorized into two main types based on how they process input data: supervised learning, which relies on labeled data sets, and unsupervised learning, which identifies patterns in unlabeled data.^63^ A schematic overview of the primary algorithms associated with each ML category is provided in Figure 5.

**Figure 5:**
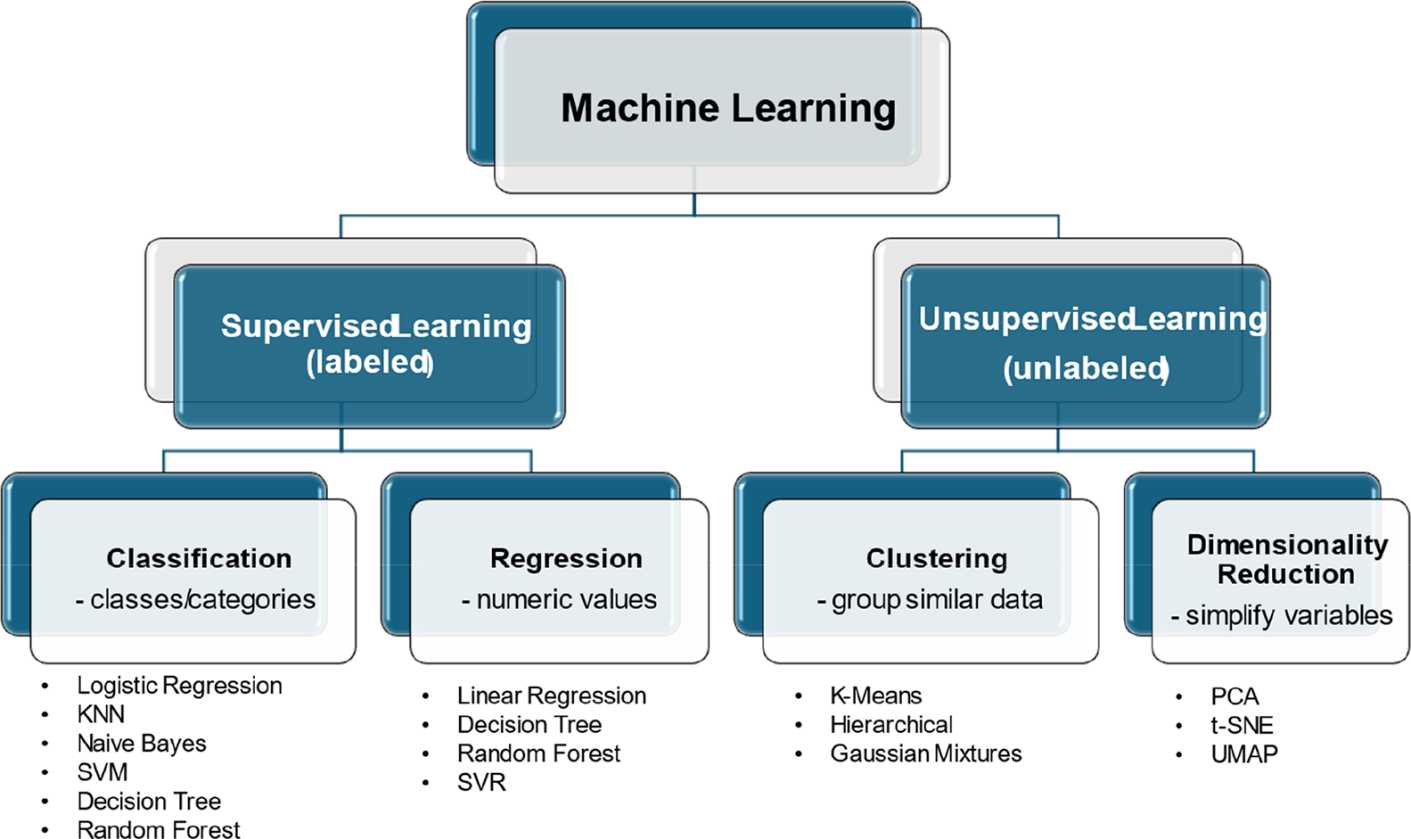
Representative diagram of the types of machine learning and the methods and algorithms used (Source: Author).

Supervised learning is a subcategory of machine learning, which consists of making AI capable of “learning” how to generate results based not only on the initial data (input) but also on the final data (output), with the entire model being built on a set of labeled data, i.e. defined by human criteria, so that AI can classify these data (so-called classification models) or make predictions (so-called regression models).^64^

As seen in Figure 5, there are many exclusive classification models (Logistic Regression, K-Nearest Neighbors, KNN, Naïve Bayes, Support Vector Machines, and SVM) when compared to those primarily used for regression, such as Linear Regression (LR). Additionally, there are also algorithms that are interchangeable between these two models [Decision Tree (DT) and Random Forest (RF)]. It should be noted that the number of available algorithms should not be considered a decisive factor when choosing between supervised learning subtypes. Instead, their suitability depends on the specific objectives and data structure of each project.^64^

## Logistic Regression

Logistic Regression works with dependent or categorical variables; i.e., the output generated by this model will always be classified binarily (e.g., “true” or “false”). One of the classic examples of this type of classification in the context of MedChem is the use of the so-called receiver operating characteristic curve, which is widely used in the context of molecular modeling (e.g., molecular docking). It is employed as a metric for evaluating the ability of a scoring function within a given docking program to discriminate between active and inactive ligands for a given pharmacological target (e.g., proteins deposited on platforms such as the Protein Data Bank (PDB). This evaluation is based on the correlation between the docking score and the experimental potency values for the same set of ligands.^65,66^

Thus, a good scoring function is one that is capable of assigning a better score, reflecting stronger ligand–protein interactions, to active or “true” molecules, typically defined by experimental potency values below a predefined threshold (e.g., IC_50_ < 10 μM), as opposed to inactive compounds with lower potency. The specific cutoff value may vary depending on the study design and intended application.^67^

## K-Nearest Neighbors

KNN is a nonparametric classification algorithm, whereby data are categorized based on the distance (usually Euclidean) between the available data. Thus, data that are closer together are scored within the same class or category. The main advantage of this learning method, compared to other classification methods, is its low processing cost and time to generate results. However, its approach is quite restricted in terms of sample space, especially for BD projects applied in MedChem. This possibly explains why none of the studies found (Table 2) used KNN, since its application is more suited to small data analyses, such as the analysis of chemical group similarity to establish a relationship between chemical structure and biological activity.^68^

## Naïve Bayes

Unlike KNN, Naïve Bayes is an algorithm based on the principle of conditional independence among features. This means that each piece of data plays an equal role in building the model, with the categorization of these data established probabilistically, in which each piece of data is classified according to the probability of belonging or not to a given class. This method is very efficient for analyzing large data sets. However, its use in BD, like KNN, is quite limited, particularly in large-scale or high-dimensional data sets. These algorithms are more commonly applied in combination with more robust methods (such as SVM) for tasks related to the generation of data restricted to textual elements, such as text search on pharmaceutical patents (Table 2)^69–71^ or evaluation of information on existing molecules (such as in the improvement of ligand-based virtual screening—LBVS) (Table 2).^72^

## Support Vector Machine

SVM is one of the best-known techniques, and this algorithm is capable of categorizing data based on distance. The focus of this algorithm is to establish a global decision boundary known as a hyperplane. This hyperplane, defined in an n-dimensional feature space, separates different classes by maximizing the margin between the closest points of each class (the support vectors). The resulting boundary aims to achieve the best possible separation between categories in the data set. Compared to the other classification algorithms already described (Logistic Regression, KNN, and Naïve Bayes), SVM offers greater flexibility and performance, particularly in handling complex data sets. Not surprisingly, some of the studies found (4 articles in Table 2) described the use of SVM in their projects, in which its application refers both to the correlation of chemical (e.g., identification of molecular patterns) and pharmacological properties (Table 2)^73^ as well as the prediction of physicochemical properties (solubility) based on experimental melting point values which when combined with molecular descriptors became a BD problem as previously discussed.^47^

## Linear Regression

LR is an exclusively predictive algorithm and therefore is not used for data classification. It is applied to identifying correlations between a dependent variable and one (simple regression) or more dependent variables (multiple regression). However, due to its limitation in handling a large number of variables (particularly in multidimensional spaces), its use ends up being more restricted in relation to other algorithms. It also requires the construction of new models for each data set.^74^

Despite these limitations, LR remains widely recognized due to its broad applicability in MedChem, especially in the construction of Quantitative Structure Activity Relationship (QSAR) models. Its continued use is also supported by the possibility of adapting the model to capture correlation, including those involving nonlinear relationships. Still, due to its limitations, the use of LR in BD applied to MedChem typically requires a combination with other types of AI techniques.^75,76^ Notably, none of the reviews reported the isolated use of this algorithm.

## Decision Tree and Random Forest

Among the types of supervised machine learning, DT and RF are among the most used algorithms by data scientists due to their flexibility. Both can be applied in the construction of classification or prediction models. As its name suggests, DT is a hierarchical, nonparametric model whose structure resembles a tree. It begins with a single set of input data (the root node) and branches into subsets (internal or decision nodes), which further divide into terminal nodes (leaf nodes) capable of representing possible outcomes based on the previous decisions.^77^

RF, in contrast, is an ensemble method based on DTs, but with more complex interconnections between nodes. It constructs multiple DTs in order to increase the model performance. The main difference lies in the operating principles of each algorithm: while DT decision-making is based on the values of each node, RF aggregates the outputs of many independently trained DTs to reach a consensus. Notably, RF is less prone to overfitting than individual DTs, making it more robust in handling noisy or high-dimensional data.^78^ If the goal is to generate clearer results, in terms of interpretability, DTs are usually the best choice. However, if the goal is to build more robust models, the use of RF is recommended.^78^ DTs are often selected for building faster and less complex models, as they are limited in working with large data sets. In contrast, RFs tend to deliver better performance in terms of accuracy, even when applied to high-dimensional data sets.^79^

Considering all of these points, it is expected that RF will be a more efficient method in BD projects applied to MedChem. Indeed, RF was one of the most cited ML approaches in the works found (Table 2), in reviews^80–83^ or original research studies.^84^

## Unsupervised Machine Learning Approach

Unsupervised machine learning refers to the use of algorithms that analyze data without prior labeling or categorization. These algorithms are typically employed for tasks such as clustering, association, and dimensionality reduction. In clustering, the algorithm identifies groupings (called clusters) based on the differences and/or similarities among unlabeled data points, with no human interference in the pattern recognition.^85^

There are several algorithms that perform clustering, with K-means (and its derivatives) and hierarchical clustering being the most widely used due to their simplicity and computational efficiency.^86^ Another widely used algorithm, particularly for dimensionality reduction, is principal component analysis (PCA). PCA is a well-established statistical method that facilitates the interpretation of complex data sets without significant loss of relevant information.^87^ More recent nonlinear dimensionality reduction techniques, such as *t*-distributed Stochastic Neighbor Embedding and UMAP (Uniform Manifold Approximation and Projection), are also frequently employed to visualize high-dimensional chemical or biological data.^88^

As with other machine learning approaches, the choice of algorithm will depend on the sample set. It is often necessary to benchmark multiple algorithms, typically using statistical metrics and visual outputs, such as cluster plots, to determine which method best captures the underlying structure of the data. These techniques are valuable for assessing chemical diversity and supporting molecular repurposing efforts.^89^

## Deep Learning Approach

Deep learning (DL) is a subcategory of AI in which all algorithms are designed to mimic the structure and functioning of the human brain. These models are built using neural networks, where data flow through interconnected layers in a manner analogous to synaptic communication between biological neurons. Technically, DL refers to neural networks with a deep architecture, typically defined as having three or more hidden layers. In the current data science scenario, including BD applications, this type of AI has become one of the most widely used in a wide range of sectors.^90,91^

There are diverse types of neural networks, namely, artificial neural networks (ANN), convolutional neural networks, and RNN (recurrent neural networks). This distinction is important: while all such models are ANNs to some extent, not every neural architecture is necessarily an ANN in a strict sense. Furthermore, different problems require different neural networks, each of which is relevant depending on the type of study. It is worth noting that all these neural networks are adaptable and can be combined with each other, as well as with supervised learning algorithms ASNN (Table 2),^47^ unsupervised methods, or even graph-based models that use data structures composed of vertices and edges (Table 2).^80^ This versatility justifies their use in the most diverse types of data analysis. Indeed, nearly half of the studies found in Table 2 reported the use of neural networks in BD projects applied to the most diverse types of approach (FBDD, LBDD, SBDD, CASD, and PDD). A broader analysis of the data summarized in Table 2 reveals patterns in the recommendation and applicability of the BD in MedChem. These trends are addressed in the last section, which suggests possible applications that tend to be more recurrent due to their relevance and feasibility.

## BD Applications for Drug Discovery in Combination with LBDD

An interesting result obtained from this survey concerns the high number of articles describing the use of BD for drug discovery in combination with LBDD. This strategy is a foundational strategy within the broader framework of computer-aided drug design (CADD).^92–94^ This statement is important because it highlights the strong connection between BD and LBDD, especially evident when classical LBDD tools, such as QSAR, are integrated with complex data types like omics data sets or large molecular databases (e.g., ChEMBL).

Notably, all of the studies described in Table 2 describe a contribution (direct or indirect) of LBDD in the construction of BD projects, particularly within the drug discovery stage. Although it is difficult to pinpoint a precise origin, many developments in BD in drug discovery can be traced back to efforts to combine data generated by combinatorial chemistry with the development of HTS techniques. The integration of these data contributes to the expansion of chemical substance libraries (whether public or proprietary) and, when complemented by experimental data on biological activity, facilitates more precise decision-making regarding the prioritization of molecules within drug discovery and development campaigns.^95,96^

Within the academic community, there has been increasing interest in the integration of HTS data with other variables such as toxicity. In this scenario, the BD approach is employed to predict the toxicity of substances from HTS assays. As a variety of platforms have already been described in previous works, we offer here a recent example: an initiative by the FDA’s National Center for Toxicological Research (NCTR). In partnership with the Center for Drug Evaluation and Research (CDER), the NCTR developed a DL-based QSAR model known as SafetAI. The objective of this model is to predict toxicity end points for promising substances that have not yet entered clinical trials. The utilization of such instruments has the potential to facilitate the selection of more secure candidates during the investigational new drug phase.^97–100^

Besides that, other AI models with the same initiative are also being developed to address related challenges. AnimalGAN is designed to reduce reliance on animal models,^101,102^ TranslatAI seeks to improve model translation or interoperability across different predictive systems,^103,104^ PathologAi focuses on the analysis of histopathological data to better understand the phenotypic effects of substances under investigation,^105^ and BERTox applies natural language processing to facilitate the extraction of toxicological information from scientific literature.^106–110^ These efforts open up new opportunities for medicinal chemists to work in an even more interdisciplinary manner in the search for new drugs.

## BD Applications for Drug Discovery in Combination with SBDD

Like LBDD, structure-based drug planning (SBDD) is one of the methodological pillars of MedChem, particularly for target-directed therapies. Because the SBDD incorporates structural information about the pharmacological target, it often allows for greater rationality and specificity in the search for new drug candidates, which has contributed to its growing prominence. However, like LBDD, SBDD has limitations, such as the dependence on experimentally determined protein structures, typically obtained through crystallography.^111^

In order to address this challenge, recent studies have explored the integration of bioinformatics and AI to model protein structures that are not yet experimentally resolved or available in structural databases, such as the PDB. These AI-driven strategies have engendered novel possibilities for structure-based design using in silico-predicted targets.^112^

A prominent example of this trend is the work led by DeepMind, which in 2018 made significant contributions through the Critical Assessment of Protein Structure Prediction (CASP) challenge, leading to the development of AlphaFold. While early versions of the system raised questions, particularly about predicting protein structures in the absence of ligand information, subsequent research demonstrated its potential to aid in the early stages of drug discovery. With the release of AlphaFold2 in 2020, the tool showed significant improvement in accuracy, establishing itself as a valuable resource in both academic and industrial settings.^112^

Despite its significant advancements, AlphaFold2 still presented some limitations, particularly in predicting protein–protein interactions and modeling protein interactions with small molecules, a central focus of MedChem. To address these challenges, recent updates have been introduced to improve the system’s ability to account for nonprotein molecular interactions, such as those involving prosthetic groups, cofactors, substrates, and ligands.^113^ These enhancements led to the development of AlphaFold3, the most recent iteration of DeepMind’s CASP initiative.^114^

The growing impact of AlphaFold on SBDD is also reflected in changes to major structural biology resources such as the PDB. The PDB has begun incorporating AI-generated structural models (referred to as computed structure models, CSM) into its database. To date, approximately 1,068,577 structures have been deposited, with 94% of these structures provided by AlphaFold.^115^ This integration of predicted structures, especially when combined with other molecular and biological data discussed earlier, has enabled the construction of new SBDD-based BD projects, contributing to the identification and prioritization of drug candidates.^115,116^

Considering these facts, the impact promoted by the creation of AlphaFold is clear, particularly when we consider the COVID-19 pandemic period, where much of the research conducted worldwide was positively impacted by the use of this tool, given its efficiency in assisting in the understanding of the pathophysiological aspects of the infection, as well as providing insight into the genetic and biochemical aspects of SARS-Cov-2.^117^ All these studies contributed to the development of vaccines, as well as to the repositioning of drugs for this disease (e.g., baracitinib).^118^ Furthermore, as mentioned earlier in the AIDD topic, the creation of AlphaFold was crucial for the discovery of new substances that are already in clinical development stages.

These findings yielded profound implications for not only medicinal chemists but also researchers across diverse areas of the natural sciences. This recognition is exemplified by the 2024 Nobel Prize in Chemistry, which was awarded to David Baker for the design and Demis Hassabis and John M. Jumper for the prediction of protein structures through computational modeling.^119^

## Conclusion and Perspectives

As demonstrated throughout this review, the use of BD and AI in rational molecule design projects contributes to the development of predictive models and the interpretation of complex biological data sets. In addition, the role of BD has recently been extended to later stages of drug development, including the regulatory phase. As the field continues to evolve, the ability to leverage and interpret large-scale data will become an essential skill set for medicinal chemists working at the interface between chemistry and biology.^120^

In this sense, MedChem, a subfield of biomedical sciences with an increasingly transdisciplinary bias, can greatly benefit from the rational use of AI to build a series of projects related to all phases of discovery, and, in particular, in the search for hits and leads, with a view to ensuring not only the maintenance of the assumptions that make up the scientific method itself but also the continuity of chemical intuition as a key element for the construction of any current MedChem project in the “era” of AI.^121^

This can be done, for example, by constructing congeneric series, which connect classic MedChem strategies (LBDD and SBDD), with cheminformatics data available for known ligands, using strategies for repositioning molecules with biological activity-based on a dialogue with information extracted from molecules containing similar structures, but which have been described for another type of activity, or even for constructing plausible hypotheses that assist in describing pharmacological results obtained in more complex trials, which lack a description of a mechanism of action at the molecular level (for cases where there is no need to conduct new experimental studies a posteriori), in addition to, of course, making use of a combination of all these strategies depending on the scientific questions to be answered in a given project. A very fruitful example of AI applications in Med-Chem projects concerns the methodological establishment of a cyclical quadrilateral of stages related to drug discovery, called DMTA (design, make, test, and analyze).^122^

This “rethinking” of data has become increasingly common in research sectors, both in industry and academia, and is a very enriching phenomenon for scientific work, since these investigations (which generate a variety of data on the same molecule or set of molecules) provide even more weight, depth, and value to prototypes that have already been discovered and contribute significantly to the design of possible new molecular entities with increasingly targeted biological activity, which aid in the search for targeted therapies. In this sense, the use of AI, when applied to chemical libraries (i.e., libraries of chemical substances with biological activity) within the context of academic research, may be another accessible approach to enhancing the drug and medicine innovation chain. This is a critical point considering the current state of medical science, which is characterized by a flood of highly complex biological data, establishing a paradigm shift in data-driven drug planning and discovery.

In addition, the provision of a variety of parameters, which are useful for establishing a relationship between chemical structure and biological activity and/or property, whose relationship needs to be increasingly accurate in order to build results that not only correlate but also show causality, ends up making the search for hits or leads increasingly challenging. This aspect needs to be taken seriously, considering the historical relationship between AI and computational modeling. In this sense, it is worth noting that, although the benefits generated by the use of AI are clear, the impact of the widespread use of these tools in all social sectors raises a series of philosophical questions that need to be discussed, especially in academia.^123^ It is no coincidence that recent efforts have been made to promote the rational and ethical use of AI in research, teaching, and extension projects, since the popularization of large language models (LLMs) has resulted in the generation of promiscuous data, which, from a scientific point of view, lacks the criteria necessary for such data to be considered meaningful.^124^ All these factors demonstrate the need for intentional dialogue on how these tools should be applied within the hermeneutics of biomedical sciences themselves, in order to ensure a lucid discussion on the nature of the method, harmonizing this apparent tension between human and AI, which has been a source of controversy since the exponential rise of ChatGPT and various other LLMs.^125^ 
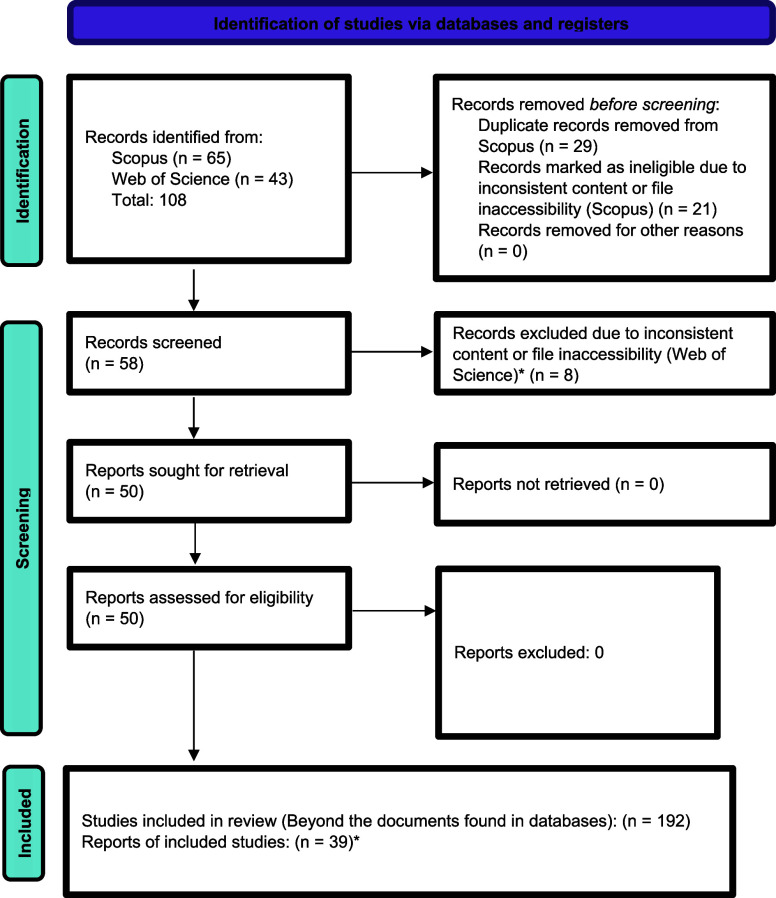


## Abbreviations

ADMET: absorption, distribution, metabolism, and toxicity
AI: artificial intelligence
AIDD: artificial intelligence drug discovery
MedChem: medicinal chemistry
BD: big data
CASD: computer-assisted synthesis design
CADD: computer-aided drug design
DDDD: data-driven drug design
DMPK: drug metabolism and pharmacokinetics
EBI,: European Bioinformatics Institute
EHR: electronic health records
RF: random forest
FBDD: fragment-based drug design
HTS: high-throughput screening
KNN: K-nearest neighbors
LBDD: ligand-based drug design
LLM: large language model
NCBI: National Center for Biotechnology Information
NIH: National Institutes of Health
NGS: next-generation sequencing
PCA: principal component analysis
PDB: protein data bank
PDD: phenotypic drug design
QSAR: quantitative structure activity relationship
ROC: receiver operating characteristic
SBDD,: structure-based drug design
SVM: support vector machine
WES: whole-exome sequencing
WGS: whole-genome sequencing
